# Universal health coverage in the context of population ageing: What determines health insurance enrolment in rural Ghana?

**DOI:** 10.1186/s12889-018-5534-2

**Published:** 2018-05-24

**Authors:** Nele Van der Wielen, Andrew Amos Channon, Jane Falkingham

**Affiliations:** 1 Centre for Research on Ageing, University of Southampton, Southampton, SO17 1BJ UK; 2 Department of Social Statistics & Demography, Social Sciences, University of Southampton, Southampton, SO17 1BJ UK; 3 ESRC Centre for Population Change, University of Southampton, Southampton, SO17 1BJ UK

**Keywords:** Ghana, Universal health coverage, Health insurance, Ageing

## Abstract

**Background:**

Population ageing presents considerable challenges for the attainment of universal health coverage (UHC), especially in countries where such coverage is still in its infancy. Ghana presents an important case study on the effectiveness of policies aimed at achieving UHC in the context of population ageing in low and middle-income countries. It has witnessed a profound recent demographic transition, including a large increase in the number of older adults, which coincided with the development and implementation of a National Health Insurance Scheme (NHIS), designed to help achieve UHC. The objective of this paper is to examine the community, household and individual level determinants of NHIS enrolment among older adults aged 50–69 and 70 plus. The latter are exempt from NHIS premium payments.

**Methods:**

Using the Ghanaian Living Standards Survey from 2012 to 2013, determinants of NHIS enrolment for individuals aged 50–69 and 70 plus living in rural Ghana are examined through the application of multilevel regression analysis.

**Results:**

Previous studies have mainly focused on the enrolment of young and middle aged adults and considered mainly demographic and socio-economic factors. The novel inclusion of spatial barriers within this analysis demonstrates that levels of NHIS enrolment are determined in part by the community provision of healthcare facilities. In addition, the findings imply that insurance enrolment increases with household expenditure even for those aged 70 plus who are exempt from the NHIS premium payment.

**Conclusion:**

Adequate and appropriate infrastructure as well as health insurance is vital to ensure movement to UHC in low and middle income countries**.** Overall, the results confirm that there remain significant inequalities in enrolment by expenditure quintile that future policy reform will need to address.

## Background

The aim of universal health coverage (UHC) is to ensure that “all people obtain the health services they need without suffering financial hardship when paying for them” [1]. Throughout the last decade, many countries have made progress towards UHC by implementing diverse policies designed to improve individuals’ access to healthcare without the fear of financial hardship [2]. On the journey towards UHC significant efforts have been made to target specific vulnerable populations including the poor, women and children [2]. However, a group of the population that is often overlooked in efforts to increase UHC is older adults. Older adults will have a large bearing on whether the goal of UHC is obtained, especially in low and middle income countries. By 2050 it is projected that, worldwide, 8 out of every 10 people aged 60 and over will live in these countries [3]. This increase in the proportion of older adults is, in turn, associated with an increasing demand for healthcare due to greater prevalence of disabilities and morbidity in older age [3].

In countries without social security systems, it is often left to the individual and their wider household to finance healthcare spending [4], potentially leading to catastrophic expenditures in times of unforeseen serious illness. Universal health *insurance* can therefore be viewed as an important component of financial protection as it aims to make healthcare affordable and accessible to all citizens. Pooled funding through health insurance can be seen as a redistribution of income, resulting in better access to services among the poorest groups through equalising the ability to pay for services. However, payments for health insurance may be problematic for older adults especially within sub-Saharan Africa, as less than one in five benefit from social security and receive a pension, while many older adults are left without a regular income [5]. Thus it is important to examine health insurance coverage as an approach to achieving UHC for this group of the population.

Ghana presents a timely opportunity for a crucial case study as it is estimated that by 2030 Ghana will have the highest percentage of its population aged 60 and over among all low and lower middle income countries in sub-Saharan Africa [6]. Furthermore, in 2005 Ghana implemented a national health insurance scheme (NHIS) aimed at achieving UHC. This paper aims to assess the reach of the Ghanaian NHIS policy to extend UHC to older adults in terms of insurance enrolment.

Previous studies that examine NHIS enrolment mainly focus on young and middle aged adults [7], or analyse all adults without differentiating older adults from their younger counterparts [8, 9]. This paper argues that it is important to consider older adults separately due to their differences in demographic and socio-economic characteristics, as well as relating to their health and disability status. Older adults suffer from higher levels of morbidity [10]. The 2010 Ghanaian Census showed that a significantly higher percentage of older adults aged 50 plus suffer from at least one disability compared to adults aged 18–49 years. Ghanaian older adults are found to be lower educated than younger adults and are predominantly living in rural areas while younger adults have tended to move into urban areas [11]. These differences in user characteristics can influence the uptake of health insurance significantly. Older adults will be relying on the NHIS in greater proportions than younger age groups, so their relationships with enrolment is crucial.

Reviewing the literature uncovers a substantial lack of studies which examine the correlates of insurance coverage among older adults living in rural Ghana. This paper aims to address this gap. The focus of the paper is on rural dwellers as this group is particularly poorly served by the Ghanaian health system [12]. Using the Ghanaian Living Standards Survey (GLSS) from 2012 to 2013, the paper examines the determinants of NHIS enrolment for 1) individuals aged 50–69 and 2) for individuals over the age of 70 and over living in rural Ghana. Individuals aged 70 plus are exempt from the NHIS premium payment and therefore the paper argues that those older adults have to be treated separately when understanding the motivations to enrol in the NHIS. Three-level multilevel models examine the importance of individual, household and community level characteristics on influencing NHIS enrolment.

There is good reason to expect that both household and community characteristics affect NHIS enrolment. Previous research has highlighted that local facility conditions, such as stocks of medication, quality of care and waiting times, are a barrier to healthcare usage and NHIS enrolment [13, 14]. Moreover, if healthcare services are not available or physically accessible, health insurance affiliation becomes devoid of value for the individual as any desired care cannot be attained. The majority of older people in Ghana live in rural areas [11] where physical accessibility of healthcare services is particularly poor [15]. In addition, previous research found that although NHIS enrolment is at an individual level, household level characteristics influence NHIS uptake [16, 17]. The household environment is expected to inform the decision as to whether to join the NHIS, with social interactions expected to mediate information about the NHIS. One household member’s positive or negative experience with the NHIS can influence the decision of another member to be part of the scheme.

### The Ghanaian National Health Insurance Scheme

The NHIS is an ambitious model that aims to protect every citizen against financial hardship if they decide to seek basic healthcare. Although it is mandatory to be part of an insurance scheme, in practice enrolment in the NHIS is seen as voluntary [18]. No penalties apply for not being a member of any insurance scheme and individuals, apart from those working in the formal labour sector, are not enrolled by default.

In order to become a member of the NHIS, individuals first need to register at the local district office and pay a registration fee, followed by a premium. The registration fee (around GH¢ 4; varies by district) is paid by everyone, irrespective of age [16]. The NHIS premium is based proportionally on people’s income with the poorest^1^ and individuals aged 70 plus being exempt from any premium fees. The official minimum payment amounts to GH¢7.20 per annum and the upper limit is not allowed to exceed GH¢48.00 ($1.60 to $10.64)^2^ [19]. However, in reality this scaled to income fee is only applied for people working in the formal sector. Those working in the informal sector pay flat rates of GH¢ 7.20 per annum [20].

The NHIS package includes outpatient services, inpatient services, oral health, eye care, maternity care and emergencies. However, the system is not responsive enough to the needs of older adults. Care within the home, hearing aids, cancer treatment (with the exception of cervical and breast cancer treatment) and dentures are excluded from the NHIS benefit package. Moreover, as stated above, in order to become a member of the NHIS individuals first need to register at their local district office. This requirement discriminates against older adults, as travel to the local district scheme office may be problematic for some due to mobility or transport issues (especially in rural areas).

## Methods

### Data

The data for the analysis was taken from the Ghanaian Living Standards Survey (GLSS) round 6 (2012–2013) – a national representative survey which included information on the living conditions and well-being of Ghanaian households. This survey sampled 18,000 households within 1200 Enumeration Areas (EA), with 16,772 households being interviewed; a 93% response rate [21]. The survey covered all household members and its aim was to collect detailed information on demographic characteristics, education, health and employment of household members. The supplementary GLSS Community Module was administered to rural communities which are clustered in the rural enumeration areas selected for the GLSS main survey, with the aim to collect additional community level characteristics. The categorisation of areas into ‘urban’ and ‘rural’ was based on census classifications. Localities with less than 5000 residents were categorised as rural [22]. The Community Module collected important information on the availability of healthcare facilities within the community, which are of particular interest for this paper. The community questionnaire was administered to chiefs and elders as well as opinion leaders. More detailed information can be found in the Community Facilities Report [23].

### Definition of variables

The GLSS survey respondents were asked whether they are registered under a health insurance scheme, followed by a question whether they currently hold a valid NHIS insurance card and are therefore enrolled. NHIS enrolment is reviewed annually and only when having a valid insurance card are individuals entitled to free NHIS services. The analysis in this paper therefore distinguishes between being currently enrolled (having a valid NHIS card and therefore entitled to free NHIS healthcare) and not currently enrolled (not having a valid NHIS card).

This paper focuses on older adults aged 50 plus. In contrast to studies defining older adults as those over 65 years [24], it is argued that in this study context the chronological definition of 65 is not appropriate. In an African context a chronological age of 50 or 55 serves as a more appropriate cut-off point when defining an older adult. In Ghana life expectancy at birth is 62 years, while healthy life expectancy at birth is 54 years [25]. Further, the population aged 50 plus is under-represented in surveys carried out in many low and middle income countries, with health surveys commonly focusing on adults up to 49 years [26].

At age 70 all individuals are exempt from the premium (although the registration fee still needs to be paid). Individuals aged under 70 still have to pay the premium. To account for these differences by age the anaysis in this paper looks at older adults aged 50–69 and 70 plus separately. To control for spatial characteristics the provision of a healthcare clinic or hospital in the community was included. It is expected that only when healthcare services are both available and accessible that health insurance uptake can be improved. In Ghana, previous research showed that long distances as well as increasing travel time to healthcare facilities reduces healthcare utilisation and NHIS enrolment [9, 27]. Older adults are disadvantaged in their access to healthcare as they predominately live in rural areas where the provision of healthcare services is limited. Large distances to healthcare facilities can be a barrier for less mobile older adults to access services. The existence of a motorable road that passes through the community was included to control for transportation barriers. UNICEF [4] examined the physical accessibility of services in Ghana, Bangladesh, Vietnam and Rwanda and found that “in many cases, even when transport was available, difficult terrain and the lack of roads, particularly in rural areas, prohibited access” (p.27). In the GLSS the existence of a motorable road or healthcare facility in the community was reported by the community chiefs in the community questionnaire.

Based on previous findings (e.g. [7, 8]) living standards have the strongest effect on health insurance enrolment. In this study this was measured through household expenditure. The GLSS provided a household expenditure index collapsed into quintiles. In order to capture expenditure accurately the GLSS used expenditure diaries where survey participants recorded their expenditure over a set period of time. Expenditure was based on the average expenditure on food, beverages, tobacco, expenditure on non-food items (including transportation) and expenditure on housing. All expenditure data were converted to daily measures, which were then summed. To account for differences in regional prices, household expenditure was adjusted to the regional costs of living in the year 2012. More detailed information can be found in the GLSS Poverty Profile report [28].

The employment status of the household head, internet access and household size were included in the analysis at the household level. Recent evidence from Ghana presented a positive association between NHIS enrolment and family size, which is most likely explained by increased feelings of responsibility [8] and free enrolment for children when both parents are a member of the NHIS. Kusi, et al. [16] showed that the yearly NHIS contributions place a burden on poorer and larger households. Internet access is hypothesised to increase the overall awareness of the NHIS.

Health status was measured through the presence of any disabilities that limit full participation in activities. A measure of health was included in order to examine whether individuals with poor health are more likely to self-select into insurance membership in order to obtain treatment. Disability, rather than self-rated health status, was used as it is possible that enrolment in the NHIS may lead to an improvement of health status, while long term disability is less likely to be affected. Marital status was included as a control variable as the literature indicated that married people in Ghana are more likely to enrol in the health insurance scheme [29–31]. In addition married couples have the opportunity to pool financial resources making it easier to afford insurance membership [7]. In addition, the educational level of the older adult, religion and ethnicity was included in the model. Osei-Akoto and Adamba [9] argue that in an African context ethnic and religious diversity determines enrolment in health insurance.

A full definition of each of these variables is shown in Table 1.

**Table 1:** Coding of used variables

	Variable	Coding
Dependent Variable	NHIS enrolment	Coded dichotomously yes – having a valid NHIS card – and no – not having a valid NHIS card
Community level variables	Hospital in community	Coded dichotomously. Reference: None
	Healthcare clinic in community	Coded dichotomously. Reference: None
	Motorable road passes through community	Coded dichotomously. Reference: None
	Region	Included the 10 regions in Ghana with Upper West used as reference
Household level variables	Standard of living	Expenditure quintile. Reference category: Poorest (1st quintile)
	Employment status of head	Converted into 4 categories: employee public, employee private, self-employed, other inactive. Reference category: employee public
	Internet access	Coded dichotomously using the category no access as reference
	Household size	Log household size
Individual level variables	Sex	Coded dichotomously using the category male as reference
	Education	Converted into 3 categories from none to secondary/ higher education with no education as reference category
	Marital status	Grouped into 3 categories: married/cohabiting, never married/separated/divorced and widowed. Reference category: married/cohabiting
	Disability	Coded dichotomously into yes - suffering from disabilities that limit his/her full participation in life activities - and no – not suffering from disabilities that limit his/her full participation in life activities. Latter was used as the reference category
	Religion	Grouped into none (reference category), Christian and Muslim
	Ethnicity	Grouped into seven categories. Reference category: Akan

### Statistical analysis

Pearson’s Chi-squared test was used for bivariate analysis of the outcome and explanatory variables. To examine the correlates of NHIS enrolment, binary logistic multilevel modelling was used. The model here consists of three levels, with individuals (level 1) nested within households (level 2) nested within communities (level 3) and hereby allowing the distinction of individual, household and community effects on health insurance enrolment. A likelihood ratio test was used to compare the use of a multilevel model over a single-level model. The likelihood ratio tests showed that a three-level model was preferred over a single-level model as well as the simpler two-level ‘individuals-within-households’ model and the two-level ‘individuals-within–communities’ model. It confirmed that household and community variance are separately significant.

A sequential model-building process was applied to understand the extent to which user characteristics, household characteristics and contextual access barriers influence participation in the NHIS. A total of three models were estimated. The first model controlled for the key community level variables including the physical accessibility of services. The second model expanded the first model and adds household level characteristics. Individual demographic characteristics were controlled for in the third model.

The analysis was restricted to complete cases of 5846 older adults (4086 aged 50–69 and 1760 aged 70 plus) in 4213 households in 640 communities.

The analysis was carried out using Stata 14 [32].

## Results

### Descriptive results

Table 2 reveals that when having *no* hospital in the community a higher percentage of older adults aged 70 plus (61%) were enrolled in the NHIS, compared to their 50–69 year old counterparts (51%). When a hospital was in the community, the gap in insurance enrolment among those two age groups decreased considerably. Table 2 also shows that there are regional differences in the NHIS coverage. For both age groups in the Upper West, Upper East and Brong-Ahafo region the insurance coverage is particularly high. Lower coverage is found in the Central and Northern region as well as Greater Accra.

**Table 2:** Insurance coverage by background characteristics

		Age: 50–69		Age: 70 plus	
Variable	Characteristics	%Insured	N	%Insured	N
Hospital in community	No	51.39	3946	60.61	1683
	Yes	78.57	140	80.52	77
	*p-value*	0.000		0.000	
Clinic in community	No	47.25	2567	55.35	1131
	Yes	60.90	1519	72.50	629
	*p-value*	*0.000*		*0.000*	
Motorable road passes through community	No	41.79	627	43.48	253
	Yes	54.24	3459	64.50	1507
	*p-value*	*0.000*		*0.000*	
Region	Upper West	65.22	716	70.67	300
	Western	49.83	303	44.64	112
	Central	34.83	333	49.49	99
	Greater Accra	38.46	52	56.52	23
	Volta	53.79	422	60.14	281
	Eastern	48.43	479	62.50	200
	Ashanti	58.17	349	67.67	133
	Brong Ahafo	58.47	313	73.64	129
	Northern	31.71	514	42.44	172
	Upper East	62.15	605	66.24	311
	*p-value*	*0.000*		*0.000*	
Expenditure	Poorest	46.38	1175	54.76	546
	Poor	49.42	775	63.12	404
	Middle	51.79	697	61.86	291
	Rich	51.91	682	64.55	299
	Richest	65.39	757	70.45	220
	*p-value*	*0.000*		*0.001*	
Employment status of head	Employee public	84.78	138	80.77	26
	Employee private	50.24	3529	59.63	1288
	Self employed	85.71	35	81.25	16
	Other inactive	56.88	385	65.12	430
	*p-value*	*0.000*		*0.012*	
Internet access	No	61.38	580	58.19	287
	Yes	50.83	3506	62.12	1473
	*p-value*	*0.000*		*0.211*	
Sex	Male	47.65	1897	57.59	837
	Female	56.37	2189	65.01	923
	*p-value*	*0.000*		*0.001*	
Education	None	48.85	2270	61.19	1430
	Primary	53.98	1595	60.73	303
	Secondary and higher	76.02	221	85.19	27
	*p-value*	*0.000*		*0.038*	
Marital Status	Married	52.53	3025	62.11	921
	Never married/Separated/Divorced	45.22	387	50.00	112
	Widowed	55.49	674	62.45	727
	*p-value*	*0.005*		*0.036*	
Disability	Yes	48.24	170	58.41	214
	No	52.50	3916	61.90	1546
	*p-value*	*0.275*		*0.325*	
Religion	None	38.46	318	52.76	163
	Christian	56.97	2461	65.63	998
	Muslim	47.09	1287	56.93	599
	*p-value*	*0.000*		*0.000*	
Ethnicity	Akan	53.14	1244	65.42	480
	Ga-Dangme	45.60	193	54.79	73
	Ewe	49.23	457	62.35	255
	Guan	52.02	198	60.61	99
	Gurma	36.63	243	37.27	110
	Mole-Dagbani	54.46	1333	61.76	578
	Other	58.85	418	67.27	165
	*p-value*	*0.000*		*0.000*	

Based on a total of 5846 observations with complete information

Results further indicate that NHIS enrolment was significantly higher in communities which had a healthcare facility or clinic (Table 2). This effect was found to be true for both age groups but was stronger for older adults aged 70 plus. Also a motorable road that passes through the community increased NHIS enrolment.

Differences in NHIS uptake were also seen by household expenditure quintile. Higher financial well-being was associated with higher NHIS uptake. A similar relationship was observed for education. More educated older adults were more likely to be enrolled.

### Multi-level regression results

Table 3 displays the odds ratios estimated from the multilevel regression analysis which examines the correlates of NHIS enrolment for older adults aged 50–69 and aged 70 plus. Model 1 indicates that the availability of a healthcare clinic in the community had a positive effect on insurance enrolment among both age groups. Moreover, it is seen that road access in the community positively influenced insurance enrolment. This effect was found to be stronger among those aged 70 plus. Model 1 further highlights that region was significantly related to enrolment in the NHIS. The likelihood of enrolling in the NHIS for older adults was highest in the Upper West Region, reflecting the enrolment patterns shown in Table 2. The addition of household level factors (Model 2) only attenuated the odds ratios for community factors slightly, while adding individual level factors (Model 3) did not have a large effect, with significance of factors enduring throughout. However the addition of household and individual factors increased the strength of the relationships with region, with those in the Central region least likely to be enrolled even when controlling for all other factors.

**Table 3:** Odds ratio of NHIS enrolment among older adults

	Age: 50–69 (*N* = 4086)												Age: 70 plus (*N* = 1760)											
Variable	Model 1				Model 2				Model 3				Model 1				Model 2				Model 3			
	Odds ratio		95% CI		Odds ratio		95% CI		Odds ratio		95% CI		Odds ratio		95% CI		Odds ratio		95% CI		Odds ratio		95% CI	
Hospital in community																								
No	1.00				1.00				1.00				1.00				1.00				1.00			
Yes	4.27	**	1.50	12.10	2.92	*	1.04	8.17	2.98	*	1.07	8.32	2.60		0.71	9.59	2.45		0.68	8.84	2.18		2.18	7.86
Clinic in community																								
No	1.00				1.00				1.00				1.00				1.00				1.00			
Yes	2.06	***	1.39	3.05	1.86	**	1.26	2.74	1.80	**	1.23	2.65	2.70	***	1.56	4.65	2.43	**	1.43	4.13	2.43	**	1.42	4.16
Motorable road passes through community																								
No	1.00				1.00				1.00				1.00				1.00				1.00			
Yes	2.72	***	1.58	4.67	2.51	**	1.47	4.29	2.31	**	1.35	3.96	4.44	***	2.12	9.30	3.77	***	1.84	7.69	3.72	***	1.81	7.66
Region																								
Upper West	1.00				1.00				1.00				1.00				1.00				1.00			
Western	0.24	**	0.11	0.54	0.10	***	0.04	0.23	0.05	***	0.02	0.15	0.14	**	0.05	0.43	0.06	***	0.02	0.22	0.02	***	0.00	0.08
Central	0.08	***	0.03	0.18	0.04	***	0.02	0.10	0.02	***	0.01	0.06	0.19	**	0.06	0.58	0.07	***	0.02	0.25	0.02	***	0.00	0.08
Greater Accra	0.15	*	0.03	0.73	0.06	**	0.01	0.31	0.03	***	0.01	0.19	0.50		0.07	3.54	0.19		0.03	1.35	0.12		0.01	1.10
Volta	0.36	**	0.18	0.73	0.18	***	0.08	0.38	0.10	***	0.03	0.26	0.39	*	0.16	0.92	0.22	**	0.09	0.55	0.11	**	0.03	0.43
Eastern	0.23	***	0.11	0.47	0.14	***	0.06	0.30	0.07	***	0.03	0.19	0.49		0.19	1.21	0.25	**	0.09	0.66	0.10	**	0.03	0.37
Ashanti	0.54		0.25	1.15	0.26	**	0.12	0.58	0.15	***	0.06	0.41	0.61		0.22	1.69	0.28	*	0.10	0.83	0.08	***	0.02	0.32
Brong Ahafo	0.60		0.28	1.30	0.34	**	0.16	0.76	0.23	**	0.10	0.56	1.50		0.52	4.28	0.91		0.32	2.60	0.42		0.12	1.38
Northern	0.07	***	0.03	0.16	0.07	***	0.03	0.15	0.07	***	0.03	0.17	0.12	***	0.04	0.35	0.11	***	0.04	0.32	0.14	***	0.05	0.42
Upper East	0.87		0.44	1.72	0.67		0.34	1.32	0.72		0.37	1.41	0.83		0.35	1.93	0.63		0.27	1.47	0.73		0.31	1.72
Expenditure																								
Poorest					1.00				1.00								1.00				1.00			
Poor					1.56	*	1.05	2.32	1.50	*	1.00	2.24					1.70		0.98	2.93	1.72		0.98	3.00
Middle					2.58	***	1.65	4.03	2.29	***	1.46	3.59					2.10	*	1.13	3.91	2.02	*	1.07	3.79
Rich					3.53	***	2.19	5.70	3.04	***	1.88	4.93					3.06	**	1.57	5.98	2.60	**	1.33	5.08
Richest					9.63	***	5.58	16.64	7.42	***	4.28	12.88					4.09	***	1.87	8.95	3.73	**	1.69	8.23
Employment status of head																								
Employee public					1.00				1.00								1.00				1.00			
Employee private					0.11	***	0.04	0.27	0.16	***	0.06	0.42					0.35		0.06	1.98	0.27		0.04	1.60
Self employed					1.30		0.17	9.89	1.33		0.17	10.49					1.57		0.09	26.77	0.77		0.04	14.29
Other inactive					0.16	***	0.06	0.43	0.22	**	0.08	0.61					0.49		0.08	2.88	0.39		0.06	2.40
Internet access																								
No					1.00				1.00								1.00				1.00			
Yes					0.73		0.45	1.20	0.72		0.44	1.20					1.69		0.87	3.27	1.47		0.74	2.93
Household size																								
Log					1.45	***	1.18	1.78	1.42	**	1.14	1.77					0.79		0.60	1.05	0.79		0.59	1.07
Sex																								
Male									1.00												1.00			
Female									2.04	***	1.58	2.64									1.86	**	1.18	2.94
Education																								
None									1.00												1.00			
Primary									1.23		0.91	1.67									0.76		0.45	1.30
Secondary and higher									3.10	**	1.58	6.10									3.33		0.48	23.28
Marital Status																								
Married									1.00												1.00			
Never married/Separated/Divorced									0.61	*	0.39	0.96									0.25	**	0.11	0.57
Widowed									0.89		0.62	1.28									0.48	**	0.28	0.81
Disability																								
No									1.00												1.00			
Yes									0.89		0.49	1.60									0.47	*	0.26	0.86
Religion																								
None									1.00												1.00			
Christian									1.97	**	1.22	3.18									2.17	*	1.09	4.31
Muslim									1.47		0.86	2.52									1.38		0.67	2.83
Ethnicity																								
Akan									1.00												1.00			
Ga-Dangme									0.97		0.44	2.12									0.24	*	0.07	0.83
Ewe									1.00		0.50	2.02									0.56		0.18	1.70
Guan									1.11		0.48	2.55									0.24	*	0.07	0.81
Gurma									0.53		0.23	1.21									0.11	***	0.03	0.36
Mole-Dagbani									0.47	*	0.23	0.92									0.15	**	0.05	0.46
Other									0.76		0.34	1.68									0.27	*	0.08	0.88
Random effects																								
Between household variance	4.64		3.17	6.78	3.96		2.64	5.94	4.02		2.68	6.03	3.77		1.69	8.40	3.50		1.52	8.05	3.52		1.52	8.15
Between community variance	2.44		1.74	3.42	2.44		1.74	3.42	2.35		1.67	3.32	2.48		1.36	4.53	2.30		1.25	4.23	2.24		1.19	4.19
Variance partition coefficient																								
Household	0.45				0.41				0.42				0.40				0.39				0.39			
Community	0.24				0.25				0.24				0.26				0.25				0.25			

***p < 0.001, **p < 0.01 *p < 0.05

For those aged 50–69, Model 1 showed that 45% of the variation in NHIS enrolment lies between households and 24% lies between communities. Similarly, for those aged 70 and over, 40% of the variation in NHIS enrolment lies between households and 26% lies between communities.

Model 2 adds household level characteristics. This shows that a higher household expenditure was associated with increased NHIS enrolment. This was found to be true even for older adults aged 70 plus who are exempt from the NHIS premium payment. Furthermore, for individuals aged 50–69 the likelihood of being insured increased when the household head was employed in the formal sector. This effect was found to not be significant when restricting the analysis to individuals aged 70 plus. Adding household level characteristics (Model 2) reduced the between household variance for both age groups.

Model 3 includes the characteristics of the individual. The analysis here showed an effect of marriage on insurance affiliation. Married older adults were more likely to be enrolled in the NHIS compared to those never married, separated or widowed. Those aged 50–69 with secondary and higher education were more likely to be enrolled compared to those without any education. No significant education effect was found for those aged 70 plus. Being Christian was significantly associated with NHIS uptake among both age groups. Those aged 70 plus with a disability were significantly less likely to enrol in the NHIS. This effect was found not to be significant when looking at older adults aged 50–69. In both models, adjusting for individual level characteristics had minimal impact on the magnitude of household level variance. This shows that there were no large differences in individual level characteristics between households.

## Discussion

The analysis presented in this paper sheds new light on key barriers to insurance enrolment among older adults in Ghana. While other studies have studied determinants of NHIS enrolment among adults, the determinants of NHIS enrolment for older adults have remained under-researched. The multilevel model allowed the determination of which individual level, household level and community level characteristics influence participation in the NHIS among older adults aged 50–69 and 70 plus. The results of this analysis have both research and political relevance. From the sequential modelling undertaken it is clear that the relationships between community, household and individual factors with insurance enrolment are independent of each other. Hence any attempts to increase enrolment can target any or all of these levels.

The objectives of this paper focused on spatial elements as key barriers to insurance enrolment among older adults in Ghana. The paper showed that insurance enrolment is related to the provision of healthcare facilities in the community, with higher enrolment in rural communities with healthcare clinics. This finding is supported by Kusi, et al. [16], who used data from three Ghanaian districts (Kwaebibirem, Asutifi and Savelugu-Nanton) and found that “significant proportions of the fully insured (72%) and partially insured (67%) households lived less than two kilometres from the nearest NHIS accredited health facilities compared to 55.5% of the uninsured. Nearly 22% of the uninsured were more than five kilometres from the nearest NHIS accredited health facilities” (p.7). Osei Adu [33] showed that distance from the nearest NHIS registration centre is significantly associated with NHIS enrolment. For older adults especially, health services need to be physically accessible, as older adults tend to be less mobile than younger adults making it impossible to travel long distances to seek care. The results showed that the effect of healthcare clinic provision and road access on NHIS uptake was stronger for those aged 70 plus compared to those aged 50–69, further indicating that this is an issue of mobility or cost. Policymakers need to become more attuned to these risks. This issue has been highlighted by the Vice President of HelpAge Ghana, Edward Ameyibor, who has urged the NHIS to react better to the needs of older adults by proving home treatment [34].

Despite scaled-to-income fees that in theory should mitigate skewed enrolment according to household expenditure, the findings imply that insurance enrolment *increases* with household expenditure. This was also found by other research (e.g. [7, 8]). Interestingly, increasing NHIS uptake with a higher household expenditure was also confirmed for those aged 70 plus, although they are exempt from the NHIS premium payment. Health insurance is argued to be a way to improve access to healthcare. However, low enrolments among the poorest indicate a failure to reach the objective of equal access.

These findings can also be explained by the ‘inverse equity hypothesis’. This hypothesis suggests that “new health interventions will initially benefit higher socioeconomic groups and widen health inequities, but if coverage increases overtime, the poor can eventually catch up and health inequities can be narrowed” ([35], p.1). Poorer people usually do not have the financial means to seek healthcare, leading to the NHIS being their only access to healthcare. However, NHIS enrolment was found to be the lowest for the poorest older adults despite the premium exemption for all core poor adults and adults aged 70 and above. Efforts need to be undertaken to ensure that all people that fall below the poverty line and are aged 70 and over are aware of the premium exemption in the NHIS to increase enrolment among the poor. Moreover, lowering or abolishing of registration fees for this group needs to be (re-)considered. Currently, enrolment is not entirely free as a registration fee is charged even for those who qualify for the premium exemption. So far there is no evidence that indigent status protects the poorest members of society. Without interventions to ensure that the poorest people can access the NHIS they will continue to face a trade-off between financing needed healthcare and the allocation of resources for other essentials like food [36]. More research is needed to determine whether registration costs form significant further hindrance to enrolment and whether these costs can be centrally funded. The literature so far mainly focuses on premium payment without considering the effect of the registration fee. Kusi, et al. [16] showed that the registration fee puts a burden on very poor households wishing to enrol children, even though they are exempt from the premium payment. This paper suggests that there is a similar deterrent effect for poor older people.

The likelihood of being insured also increased among older adults aged 50–69 living in households where the head is employed in the formal sector. It is challenging to increase NHIS membership in the informal sector as there are no formal procedures in place to check insurance status among the self-employed. Only people working in the formal sector are authoritatively required to enrol in the NHIS, while in the informal sector no formal penalties apply for not being part of a health insurance scheme.

The positive relationship between education and enrolment for those aged 50–69 underlines the findings of Ayitey, et al. [8] who showed that for adults in general, rising education increase NHIS enrolment. This again may be evidence of the inverse equity hypothesis, with the more educated most likely to benefit from new health interventions. Further analysis showed that less than 0.5% of GLSS survey participants were not aware of the NHIS, so the education variable is not simply a proxy for knowledge of the system. Further investigation needs to be undertaken to understand why there are these differences by education in enrolment.

Amongst those over the age of 70, those with a disability are less likely to be enrolled than their non-disabled counterparts. This may indicate that access to either services or the registration process is affecting enrolment amongst this group. Disability is defined as having limitations in full participation in life activities, which may cover a wide range of issues. However, it could be hypothesised that those with a disability would be more likely to enrol in the NHIS for the available benefits, especially for those where the premium is free. The indication that the reverse is true highlights the marginalisation of this group and may indicate that different approaches are needed to engage them within the NHIS.

When looking at gender, the results show older females were more likely than males to be enrolled in the NHIS. These results of are in line with findings of Dixon, et al. [7] and Ayitey, et al. [8] who also found that females are more likely to enrol in the NHIS than males. This may be due to gender cultural roles. Dixon, et al. [37] argue that women in Ghana tend to take responsibility for the health and wellbeing of the family and thus are more likely to be aware of the benefits provided by health insurance coverage.

### Limitations

One of the limitations of this work is that it was not possible to control for all factors that potentially influence insurance enrolment, most obviously relating to the quality of the healthcare services. Omitted variable bias can occur by omitting variables that potentially influence NHIS affiliation [17]. With this case of omitted variable bias, it is expected that the effect of other explanatory variables will be overestimated [17]. A positive association between the quality of service and healthcare utilisation is expected. Although there is a common agreement in studies focused on an African context that wealth is the main driver of insurance enrolment [38], in Guinea the poor quality of services forms the main explanation of low enrolment in the Mutual Health Organisation community insurance scheme [39]. Poor healthcare quality could lead to a scenario where wealthy people self-select themselves into a private insurance system instead of choosing to be part of the NHIS as they expect better services if they go private. Other factors that were not controlled for include the availability of drugs at facilities. Gobah and Liang [40] argue that low coverage of drugs in the NHIS reduces the likelihood of individuals joining the system.

## Conclusion

The NHIS in Ghana has been described as the most important driver towards UHC in the country [36]. The goal of the NHIS is to improve access to and utilisation of healthcare in order to provide affordable healthcare to Ghanaian citizens. Rapid population ageing combined with an increasing demand for healthcare due to the greater occurrence of disabilities in older age have created an imperative for this research agenda as a means to inform policymaking. This study has highlighted that barriers in the decision to enrol in the NHIS exist. Even in the best performing communities over a third of older adults are not covered by the NHIS. Specifically, distance to healthcare facilities and household expenditure are associated with NHIS enrolment.

This paper concludes that improvements to local infrastructure are a necessary component of the implementation of a national health insurance scheme and therefore is vital to ensure movement to UHC in low and middle income countries. Residential differences in NHIS coverage require a targeted policy response and innovative insurance devices to be considered within schemes including reimbursed transport for those who live in remote areas.

In rural areas especially, NHIS enrolment rates need to be improved. In many lower and middle-income countries older adults rely on traditional institutions, such as family, as caregivers due to a lack of institutional frameworks that successfully manage health care needs of older adults [41]. However, due to the rural-urban migration of younger adults in Ghana, many older adults are left behind in rural areas.

The pro-rich bias in NHIS enrolment shows that the NHIS has failed to an extent to meet the needs of the poorest and is not a financially affordable option for the poor, although, scaled-to-income fees exists and people aged 70 plus are exempt from the NHIS premium. It is unclear whether older adults are actually aware of the premium exemption at 70 years of age due to a lack of studies that have examined this issue. Further research on this aspect is needed. Awareness of the NHIS premium exemptions could be improved through increased publicity of the system, potentially at markets or community hubs. Further community trust in the system can lead to the encouragement of community members to join the NHIS.

## Abbreviations

EA: Enumeration area
GLSS: Ghana Living Standards Survey
NHIS: National Health Insurance Scheme
UHC: Universal Health Coverage
WHO: World Health Organization

## Footnotes

1 All people who cannot show a source of income and have no fixed residence are classified as the ‘core poor’ and are in theory exempt from the premium payment.

2 Exchange rate January 2018: 1 Ghanaian Cedi = 0.22 US Dollar.
